# Anti-aging activities of an ethanolic extract of *Lycium ruthenicum* in *Caenorhabditis elegans* based on metabonomic analysis

**DOI:** 10.3389/fphar.2025.1498280

**Published:** 2025-02-27

**Authors:** Boya Cui, Lanying Liu, Xinmeng Qiao, Tao Shi, Min Yin, Shu Xu, Xu Feng, Yu Shan

**Affiliations:** ^1^ Jiangsu Key Laboratory for the Research and Utilization of Plant Resources, Institute of Botany, Jiangsu Province and Chinese Academy of Sciences, Nanjing Botanical Garden, Memorial Sun Yat-Sen, Nanjing, China; ^2^ National Wolfberry Engineering Research Center, Institute of Wolfberry Engineering Technology, Ningxia Academy of Agriculture and Forestry Sciences, Yinchuan, China

**Keywords:** dietary restriction, glycine, serine, and threonine metabolism, lifespan, nematode, spermidine

## Abstract

The fruits of *Lycium ruthenicum* Murr. (*Solanaceae*) are employed in ethnomedicine and used as a functional food. Their antioxidant, anti-aging, and hypolipidemic activities have been investigated in modern research. This study indicated that the ethanolic extract of the fruits of *L. ruthenicum* Murr. (LRM) improved oxidative and heat stress tolerance, reduced the accumulation of lipofuscin, and retarded the aging process in *Caenorhabditis elegans* (*Rhabditidae*). Furthermore, the pharyngeal pumping rate and body length decreased under LRM treatment. Moreover, metabolomic analysis and the DPClusO algorithm revealed that LRM regulated a series of lifespan-related pathways centered on glycine, serine, and threonine metabolism. These results suggest that LRM prolongs the lifespan of *Caenorhabditis elegans* via dietary restriction. Moreover, feruloyl putrescine, a kind of polyamine, was found in differential metabolites, which may be the metabolite of caffeoyl-spermidine in LRM. These findings from this exploratory study offer a new insight into the roles of *L. ruthenicum* in anti-aging activity as a functional food.

## 1 Introduction

Aging is defined as a time-dependent decline in physiological integrity, resulting in impaired functional ability and decreased quality of life (Cai et al., 2022). Despite advancements in modern medical science increasing life expectancy, the challenge remains to achieve good health alongside a longer lifespan (Beard et al., 2016). Natural remedies are widely mentioned in ethnomedical information that emerges as a valuable source for discovering remedies for age-related diseases (Wahid et al., 2020).

*Lycium ruthenicum* Murr., a perennial thorny shrub plant of the *Lycium* L. genus in the Solanaceae family, can be found in northwestern China, Central Asia, and the Caucasus of Europe. In accordance with the records of traditional Tibetan medicine classics *Si Bu Yi Dian* and *Jing Zhu Ben Cao,* the fruits of *Lycium ruthenicum* have been used for addressing diseases of the aged such as heart diseases and menopausal symptoms (Wang et al., 2018). Moreover, in Uighur medicine, they have been described as a nourishing tonic for improving eyesight, antihypertensive, and strengthening (Tao et al., 2022). *Lycium ruthenicum* fruits contain abundant anthocyanins, a class of flavonoids that possess antioxidant, anti-inflammatory, hepatoprotective, and renal protective activities, as well as immunity-enhancing effects (Zhang et al., 2019; Yu et al., 2023). In our previous study, the ethanolic extract of *L. ruthenicum* Murr. fruit (LRM) has been shown to possess potential activities in restoring spatial memory and cognitive function in mice treated with D-Galactose (D-Gal) inducing oxidative stress (Cui et al., 2023). Furthermore, *L. ruthenicum* anthocyanins have been shown to alleviate D-Gal-induced liver injuries through relieving inflammation and reversing the abnormal amino acid metabolome in aging rats (Chen S. S. et al., 2022). Its leaves are a byproduct of *L. ruthenicum* and contain flavonoids, anthocyanins, and polysaccharides (Tao et al., 2022; Chen et al., 2024; Liu et al., 2016). In traditional, leaves are used as a diuretic agent (Sharma et al., 2022). In a previous study, a water-soluble polysaccharide LRLP4-A isolated from the leaves could significantly increase macrophage proliferation and phagocytic ability in RAW246.7 cells (Liu et al., 2016).

*Lycium ruthenicum* extract has been demonstrated to prolong the lifespan of *Caenorhabditis elegans* in collaboration with the IIS pathway via sir-2.1 and hsf-1, but independent of daf-16 (Xiong et al., 2021). Metabolomic analysis contributes to discovering potential aging and age-related disease biomarkers (Parkhitko et al., 2020). Anthocyanins in *L. ruthenicum* have been reported to decrease oxidative stress, aging-related liver injury, and reverse abnormal metabolomic of the hippocampus and serum in aging rats (Chen S. S. et al., 2022; Chen S. et al., 2022). However, there remains a gap in metabolomic studies related to *C. elegans*. This study aims to address this gap by investigating the anti-aging activity of LRM in *C. elegans*. The effects of LRM on lifespan, lipofuscin level, resistance to oxidative and heat stresses, and other physiological indices in *C. elegans* were explored, with the objective of elucidating the anti-aging potential of LRM. Furthermore, the alteration of metabolites in *C. elegans* after being treated with LRM was analyzed.

## 2 Materials and methods

### 2.1 Chemicals and reagents

Ethanol was purchased from Saifurui Technology Company (Tianjin, China). 5-Fluorouracil (5-FU), tripotassium citrate, spermidine, levamisole hydrochloride, and paraquat (PQ) were purchased from Macklin Biochemical Technology Company (Shanghai, China). MgSO4, CaCl2, K2HPO4, KH2PO4, NaCl, and sucrose were purchased from Hushi Laboratory Equipment Company (Shanghai, China). Cholesterol was purchased from Yuanye Bio-Technology Company (Shanghai, China). Tryptone and yeast extract were purchased from Oxoid (Hampshire, United Kingdom). Agar powder was purchased from Solarbio Science and Technology Company.

### 2.2 Plant extract preparation

*Lycium ruthenicum* was identified by Dr. Mei Tian at Institute of Botany, Jiangsu Province and Chinese Academy of Sciences (Nanjing, China). The fruit samples (lead <0.5 ppm, Pymetrozine < 2 ppm.) were selected from the barbary wolfberry planting base of Barbary Wolfberry Engineering Technology Research Institute, Ningxia Academy of Agriculture and Forestry Sciences (Ningxia China) in August 2022. The sample of dried fruit (HGGQG20220723) was reserved in the herbarium of Institute of Botany, Jiangsu Province and Chinese Academy of Sciences (Nanjing, China). The method of extraction and analysis was performed as reported previously (Cui et al., 2023). In short, the dried fruits were extracted with 70% ethanol at 45°C. The extracted liquid was applied to an Amberly (1 kg) column. Then, 5% formic acid was used to remove the Saccharides. The EtOH-H2O-HCOOH (50:50:5) was used to collect the phytochemicals. After the evaporative concentrating of the extracting solution, the last extract collected was named *L. ruthenicum* extract (LRM).

Agilent 1260 UPLC-DAD-6530 ESI-QTOF-MS equipped with a Poroshell 120 SB-Aq C18 analytical column (4.6 mm × 100 mm, 2.7 μm, Agilent, United States) was employed for analyzing the phytochemical metabolites of LRM. Liquid chromatography conditions are as follows. The mobile phase composition: 1% formic acid in water (A) and acetonitrile (B); the gradient elution program: 0 min, 10% B; 35 min, 15% B; 40 min, 20% B; 50 min, 100% B; sample size: 10 μL; flow rate: 0.5 mL/min; column temperature: 40°C; detection wavelength: 535, 280, 254 nm. The mass spectrometry conditions are as follows. ESI ion source mass spectrometry detector, positive ion mode (100–3,200 m/z); nebulizer pressure 50 psi; the drying gas flow rate: 10 mL/min; the drying gas temp: 350°C; the capillary voltage: 4000 V; fragmentor voltage: 205 V; the MS/MS collision energy: 35 V.

### 2.3 Preparation of *Caenorhabditis elegans* strains

The *Caenorhabditis elegans* strains (N2) and *E. coli* OP50 used in this study were obtained from *Caenorhabditis* Genetics Center. *Caenorhabditis elegans* was raised on plates of Nematode Growth Medium (NGM) or S medium seeded with *E. coli* OP50 and maintained at 20°C. Nematodes that exploded, moved to the wall of the plate, and drilled into the medium were eliminated from further data analysis.

### 2.4 LRM preparation and treatment

The LRM was dissolved in ddH2O to make a stock solution with a concentration of 10 mg/mL. LB liquid medium containing OP50 (100 μL) was daubed onto the central surface of NGM plates (60 mm), covered with 100 μL of different concentrations of LRM solutions (ddH2O served as the control), and left overnight at 37°C. Unless otherwise noted, experimental plates with adult nematodes contained 0.07 mg/mL of 5-FU to prevent the production of progeny.

### 2.5 Lifespan and mobility analysis

The specific quantity number of synchronous L4-stage nematodes were transferred onto NGM plates containing OP50 and the LRM concentrations (5 μg/mL, 50 μg/mL, 100 μg/mL, 500 μg/mL, or 1,000 μg/mL), water, or 50 μg/mL spermidine (Spd). A gentle prod with a picker was used to check the survival of the nematodes. And dead individuals were counted daily.

The mobility of nematodes was measured at different cohort levels. The movement of the nematodes was classified into four grades: those that could move with sinusoidal motion autonomously were defined as grade A, those that needed external stimulation to help them creep were defined as grade B, those that only moved their head with a slight prod were defined as grade C, and dead individuals were defined as grade D.

The surviving individuals were moved to new plates every 48 h. All assays were performed in triplicate, with 50–70 individuals per group.

### 2.6 Determination of progeny production and generation growth assay

The synchronized L4-stage nematodes were separately transferred to individual NGM plates without 5-FU, and nematodes were transferred to new plates every day throughout the spawning period. In order to analyze the larval growth conditions, the original plates were maintained to quantify the number of progenies that normally hatched from the eggs. At least 10 nematodes were used in each treatment of this experiment.

### 2.7 Determination of pharyngeal pumping rate

The synchronized L4-stage nematodes were transferred to NGM plates containing 0 μg/mL, 50 μg/mL, 100 μg/mL, or 500 μg/mL LRM. Pharyngeal pumps per minute were determined with a handheld tally counter on day 3, day 6, and day 9. The assays were conducted for 5 nematodes per group and implemented in triplicate.

### 2.8 Determination of body length and lipofuscin level

After 10 days, 10 nematodes treated with 0 μg/mL, 50 μg/mL, 100 μg/mL, or 500 μg/mL of LRM were placed onto a 1% agarose-coated microslide. Then, they were anesthetized with levamisole hydrochloride (7 mM). The lipofuscin fluorescence intensity was detected with fluorescence microscopy at GFP channel. The lipofuscin fluorescence intensity and body length of *C. elegans* were assessed with the ImageJ software. Each treatment was implemented three times.

### 2.9 Oxidative and heat stress tolerance assay

*Caenorhabditis elegans* individuals at L4-stage were moved to the NGM plates containing 0 μg/mL, 50 μg/mL, 100 μg/mL, or 500 μg/mL of LRM, and kept at 20°C for 6 days. Nematodes were transferred to the control or appropriate LRM plates that also contained 70 mM of Paraquat dichloride for the oxidative stress tolerance assay. Mortality was scored every 12 h until all the individuals were died. As part of the heat stress tolerance assay, the nematodes were maintained at 35°C for 4 h, and the plates were maintained at 20°C subsequently. The survival rate was scored after 12 h. The experiments were conducted three times, with at least 50 nematodes per group.

### 2.10 Metabolic profiling by UHPLC-MS

The synchronized L4-stage nematodes were transferred to the liquid medium containing 0 or 100 μg/mL of LRM and kept at 20°C for 48 h (n = 3). The Vanquish UHPLC system connected with an Orbitrap Q Exactive TMHF-X mass spectrometer (Thermo Fisher, Germany) was employed to analyze the metabolite extraction of *Caenorhabditis elegans*. In short, *Caenorhabditis elegans* (100 mg) was separated from a liquid medium containing OP50. Then, these samples were fully ground with liquid nitrogen and the homogenate was resuspended in 500 μL of precooled 80% methanol with a vortex generator. The homogenate was incubated on ice for 5 min and then centrifuged at 15,000 g and 4°C for 20 min. The supernatant was diluted to a final methanol concentration of 53% with LC-MS grade water. After that, the samples were centrifuged at 15,000 g and 4°C for 20 min. Samples were injected into a Hypersil Gold column (100 × 2.1 mm, 1.9 μm). Liquid chromatography conditions are as follows. The composition of eluents for the positive polarity mode: eluent A (0.1% FA in water) and eluent B (methanol); the composition of eluents for the negative polarity mode: eluent A (5 mM ammonium acetate, pH 9.0) and eluent B (methanol). The solvent gradient: 2% B, 1.5 min; 2%–85% B, 3 min; 85%–100% B, 10 min; 100%–2% B, 10.1 min; 2% B, 12 min; flow rate: 0.2 mL/min; the Q Exactive TMHF-X mass spectrometer was operated in positive/negative polarity mode with conditions as follows. Spray voltage: 3500 V; capillary temperature: 320°C; sheath gas flow rate: 35 psi; aux gas flow rate: 10 L/min, S-lens RF level: 60; Aux gas heater temperature: 350°C.

### 2.11 Data analysis

The metabolites were annotated using the KEGG database (https://www.genome.jp/kegg/pathway.html), LIPIDMaps database (http://www.lipidmaps.org/), and human metabolome database (HMDB) database (https://hmdb.ca/metabolites). PCA and PLS-DA were performed using SIMCA 14.1. The functions of these metabolites and metabolic pathways were studied using the KEGG database.

The R-package “psych” was used to perform Pearson rank correlation analysis for all pairwise comparisons among individual metabolites. The R-package “igraph” was used to calculate network statistics. DPClusSBO 1.1 was used to identify co-accumulated metabolite groups and visualize the network (Karim et al., 2021). We performed simple graph clustering with the following settings: density value (D) was set to 0.9, cluster property (CP) was set to 0.5, minimum cluster size was set to 5, and overlapping coefficient (OV Coff) was set to default value 0.25 (Liu K. et al., 2017).

Data analyses and calculations were carried out using Graphpad Prism 8.0.2 (La Jolla, CA, United States). One-way ANOVA with Tukey’s multiple comparisons test was used to determine significance between groups. p < 0.05 was considered statistically significant.

## 3 Results

### 3.1 Phytochemical profiles of LRM

The phytochemical metabolites of LRM were analyzed using UPLC-Q/TOF-MS analysis, which revealed that the main phytochemicals in LRM were anthocyanins and spermidines (Supplementary Figure S1; Supplementary Table S1). These metabolites have been previously reported to possess anti-aging and antioxidant properties (Cui et al., 2023). Therefore, this study aims to investigate and determine the anti-aging effects of LRM in *C. elegans*.

### 3.2 LRM prolonged *Caenorhabditis elegans* lifespan and promoted mobility

**FIGURE 1:**
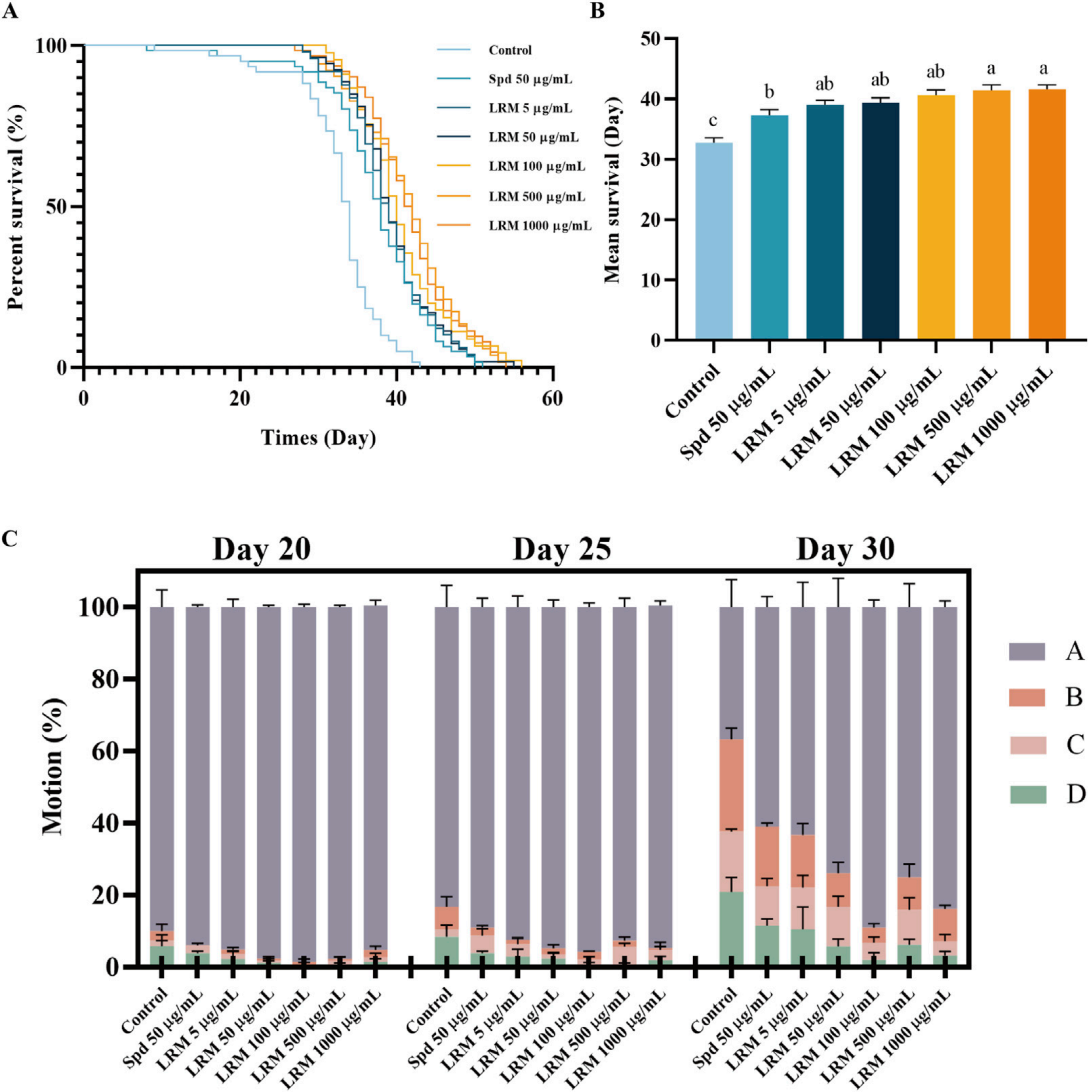
Effects of LRM on prolonging the lifespan and promoting mobility of *Caenorhabditis elegans*. **(A)** The survival curves of *Caenorhabditis elegans*; **(B)** The Average lifespan of *Caenorhabditis elegans*; **(C)** The motility of *Caenorhabditis elegans* at different stages. Data are expressed as mean ± SEM (n = 3 per group). Values with different letters in each column were significantly different (*p* < 0.05). The differences were tested by using Tukey test and one-way ANOVA.

Compared with the control group, LRM extended the N2 lifespan between 19.19% and 26.79% within the dose range of 5–1,000 μg/mL. The effect of LRM at 50 μg/mL was better than that of the anti-aging reagent spermidine at 50 μg/mL (Figures 1A, B; Supplementary Table S2).

The movement of *C. elegans* at different stages of the life cycle was conducted to determine the effect of LRM on the entire life cycle (Figure 1C). On the 30th day, in the control group, the proportion of motion Grade A nematodes decreased from 90.05% to 36.77%, while that in the other groups remained greater than 60%. In the group of LRM at 100 μg/mL, the proportion of motion Grade A individuals was 89.04%. This result indicates that treatment of LRM prolonged the lifespan of *C. elegans*, moreover, these individuals exhibited better autonomous locomotion at their middle-late life stages. Comparing the treatments of 500 μg/mL and 1,000 μg/mL LRM, the prolong rates of these two groups were 26.40% and 26.79%, respectively. Thus, we assayed the effects of LRM using only the 50, 100, and 500 μg/mL doses in all subsequent experiments.

### 3.3 Effect of LRM on progeny production and incubation of *Caenorhabditis elegans*

**FIGURE 2:**
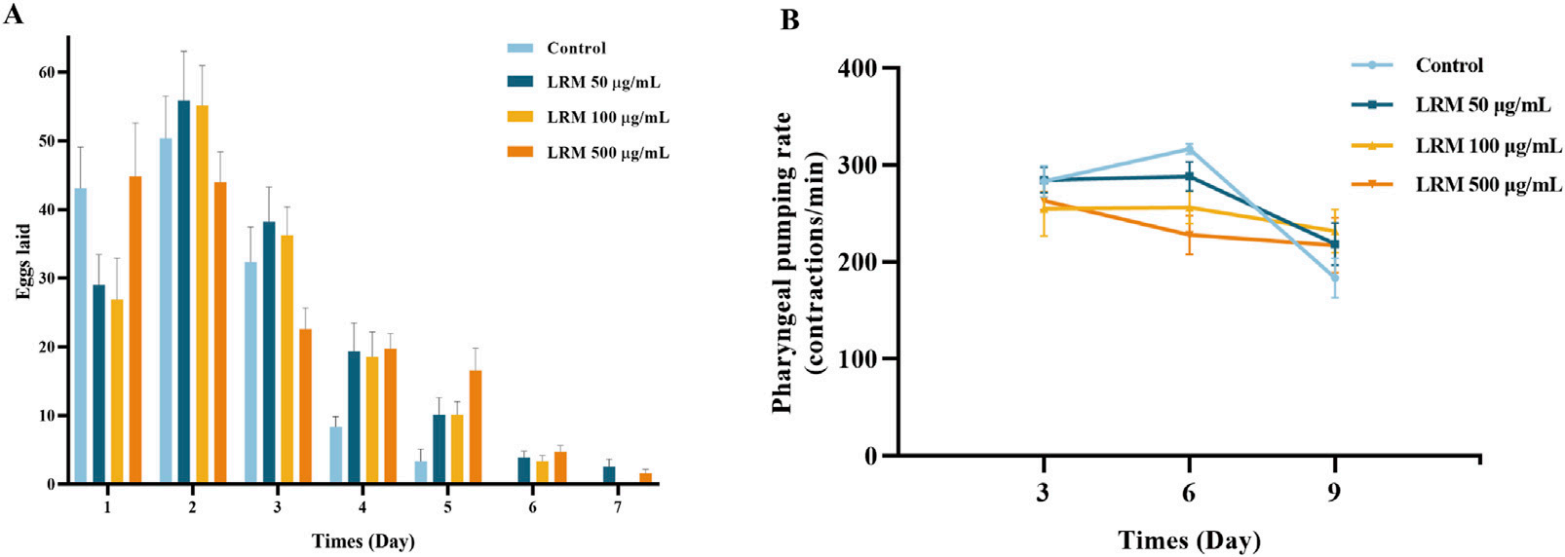
Effects of LRM on the production and the pumping rate of *Caenorhabditis elegans*. **(A)** The production per day and the reproduction period of *Caenorhabditis elegans*. Data are expressed as mean ± SEM (n = 10 per group); **(B)** The pumping rate of *Caenorhabditis elegans* during different periods. Data are expressed as mean ± SEM (n = 5 per group). The differences were tested by using Tukey test and one-way ANOVA.

*Caenorhabditis elegans* were treated with low, medium, and high doses of LRM to explore the effects of LRM on reproduction and incubation. LRM extended the reproduction period from 5 days to 7 days (Figure 2A), a characteristic associated with longer lifespans in individual nematodes (Scharf et al., 2021). Notably, 97% of the generation of each group had hatched on time, indicating no effect of LRM treatment on incubation (Supplementary Figure S2).

### 3.4 LRM affected the pumping rate of nematodes

In the early periods (day 3), the pumping rate in nematodes supplemented with LRM at 100 μg/mL and 500 μg/mL had declined compared with the control group. In the middle stage, nematodes treated with different concentrations of LRM all showed a downward trend in pumping rate in comparison with the control group. However, on the 9th day, LRM improved the pumping capacity compared with the control group (Figure 2B). Therefore, we conjectured that, in the early stage, treatment with LRM could stimulate the dietary restriction (DR) pathway. Meanwhile, as time progressed, the physiological condition of the nematodes in the control group deteriorated more than those in the LRM treatment groups.

### 3.5 LRM reduced the accumulation of lipofuscin and shortened the body length of *Caenorhabditis elegans*

**FIGURE 3:**
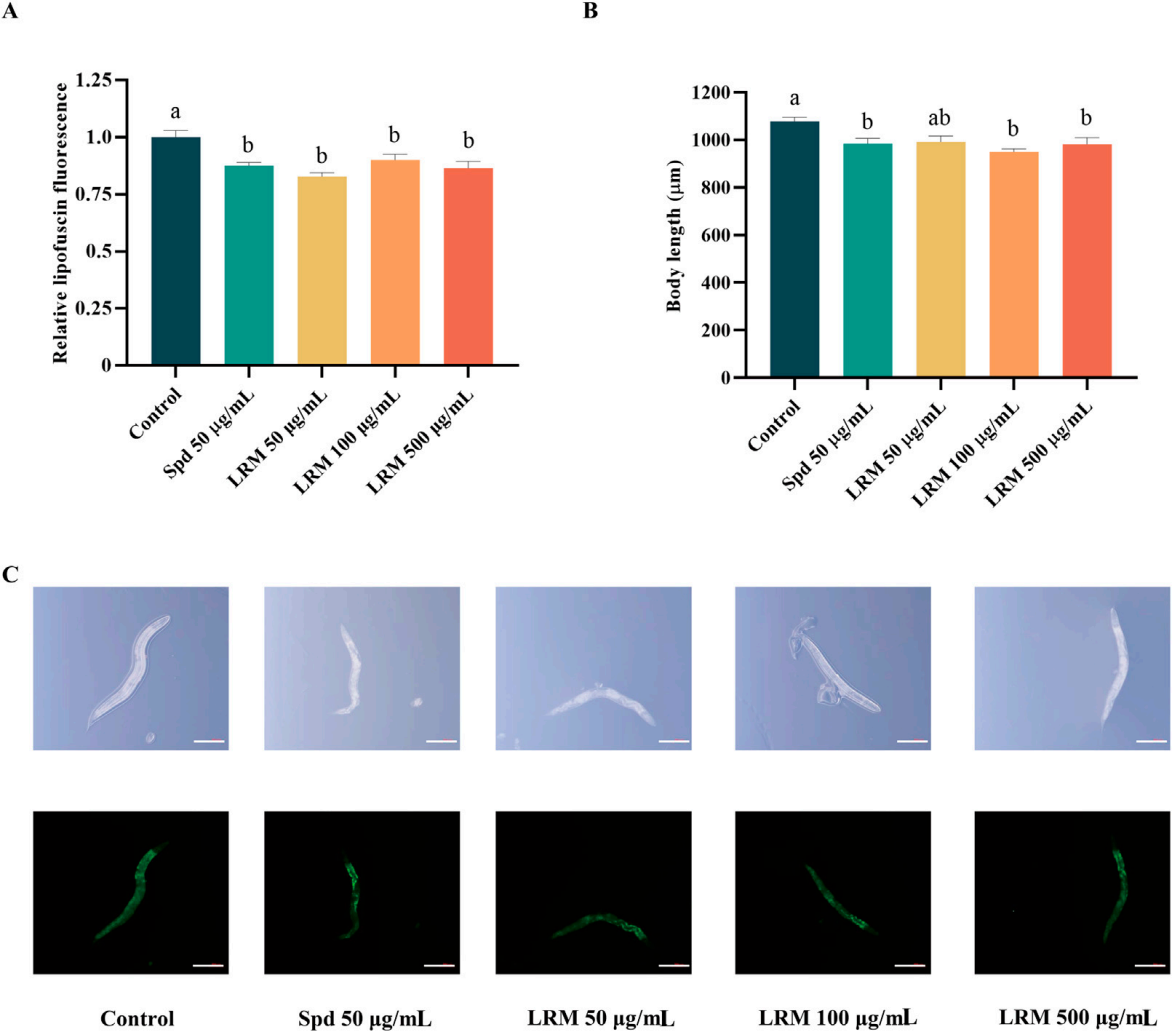
Effects of LRM on the accumulation of lipofuscin and the body length of *Caenorhabditis elegans*. **(A)** The accumulation of lipofuscin in *Caenorhabditis elegans*; **(B)** The body length of *Caenorhabditis elegans*; **(C)** The image of *Caenorhabditis elegans* (Bar = 200 μm). Lipofuscin intensity and body size were analyzed with ZEN 3.3 software. Data are expressed as mean ± SEM (n = 3 per group). Values with different letters in each column were significantly different (*p* < 0.05). The differences were tested by using Tukey test and one-way ANOVA.

Auto-fluorescent lipofuscin accumulates with the age of *Caenorhabditis elegans* (Lin et al., 2023). The measurement of lipofuscin accumulation is essential to assess aging processes. Compared with the control group, the relative lipofuscin fluorescence of *Caenorhabditis elegans* was diminished under the 50, 100, and 500 μg/mL doses, yielding 17.20%, 9.90%, and 13.58%, respectively (Figures 3A, C). This result suggested that LRM-treated nematodes maintained a more youthful state. Additionally, nematodes in a DR state, are characterized by low-age pigment fluorescence (Gerstbrein et al., 2005). The body length of the nematodes was also recorded, which was shortened partly by treatment with LRM in 50, 100, and 500 μg/mL doses (by 8.07%, 11.93%, and 9.05%, respectively) (Figures 3B, C).

### 3.6 Effect of LRM on oxidative and heat stress tolerance of *Caenorhabditis elegans*

**FIGURE 4:**
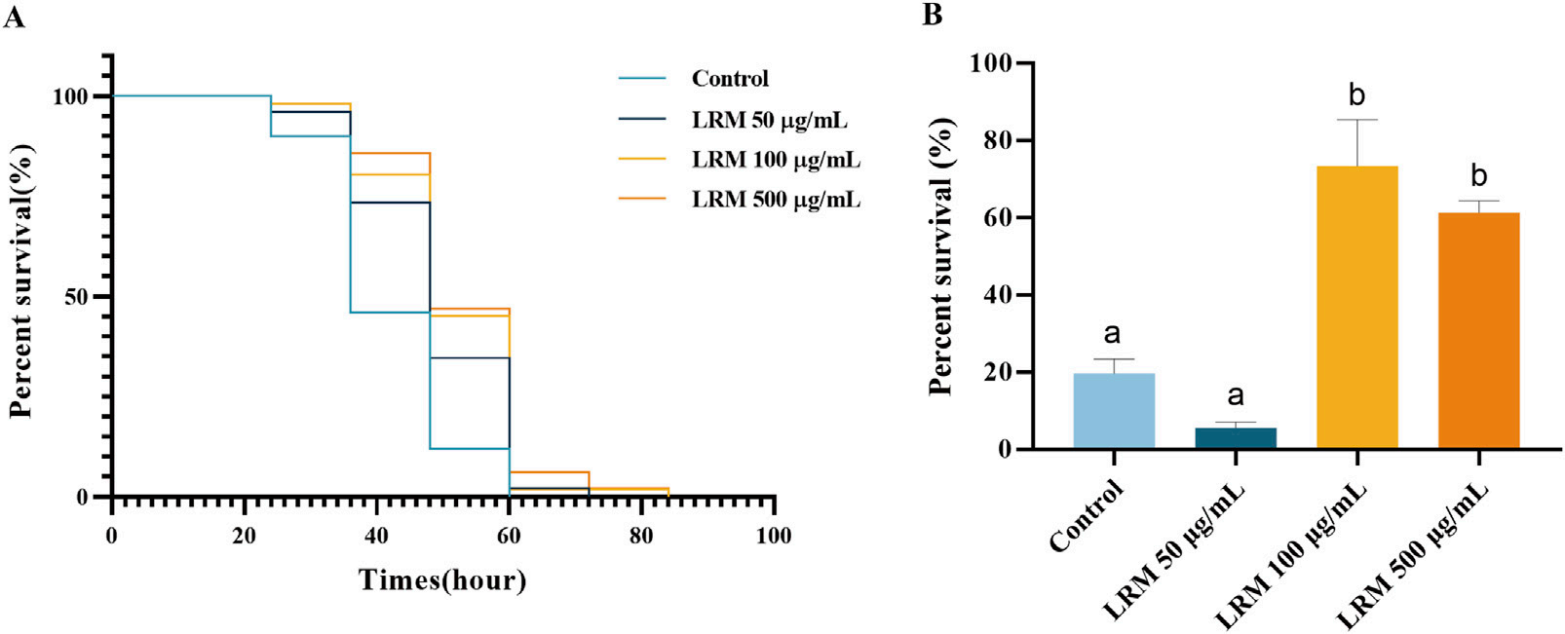
Effect of LRM on oxidative and heat stress resistance of *C. elegans*. **(A)** The survival curves of *Caenorhabditis elegans* under the oxidative and stress; **(B)** The percent survival rate of *Caenorhabditis elegans* under the heat stress. Data are expressed as mean ± SEM (n = 3 per group). Values with different letters in each column were significantly different (*p* < 0.05). The differences were tested by using Tukey test and one-way ANOVA.

Two methods were selected to explore the stress resistance of *Caenorhabditis elegans* individuals treated with LRM. PQ has been used as an oxidative stress-inducing agent. The nematodes that were fed with LRM showed an ability to resist the oxidative stress induced by PQ. The life curves moved rightward with LRM treatment. The 50, 100, and 500 μg/mL LRM extracts resulted in 16.69%, 22.82%, and 26.08% of increments in tolerance, respectively (Figure 4A). After heat stress treatment at 35°C for 4 h, the survival rates of the 100 and 500 μg/mL groups were 73.38% and 61.31%, while it was only 19.60% in the control group (Figure 4B). These results indicated increasing tolerance of *Caenorhabditis elegans* to external oxidative and heat stress after LRM treatment, which further supported the correlation between stress resistance and prolonged lifespan (Amrit and May 2010).

### 3.7 Metabolomic analyses

#### 3.7.1 Metabolites multivariate analysis

Principal component analysis (PCA) was conducted to cluster similar data (Supplementary Figure S3A). Partial least squares discriminant analysis (PLS-DA) model was constructed to better visualize the metabolite variations between the control and LRM groups, which revealed the degree of separation and indicated different metabolic characteristics [R2Y (cum) = 0.991, Q2 (cum) = 0.857] between the control and LRM groups (Supplementary Figure S3B). In the PLS-DA model, the intercept between Q2 regression line and *Y*-axis was a negative correlation, as well as the value of Q2, was lower than the value of R2, indicating that the result was effective [R2 = (0.0, 0.95), Q2 = (0.0, −1.73)] (Supplementary Figure S3C). The metabolic datasets were investigated in further analysis, based on the above results.

#### 3.7.2 Differential metabolite analysis

**FIGURE 5:**
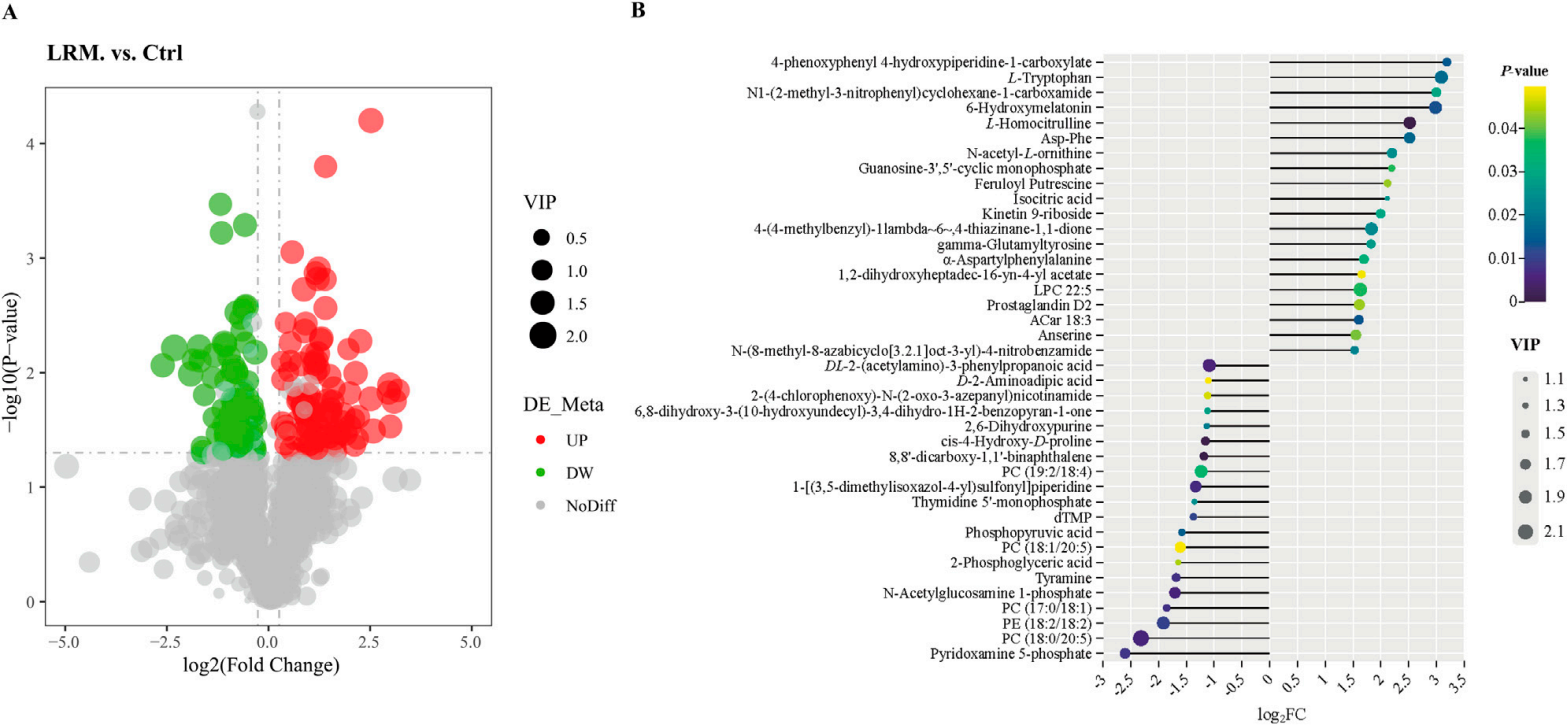
Differential metabolites between the control and LRM on *Caenorhabditis elegans*. **(A)** Differential metabolite volcano plot between the control and LRM. The dot sizes represent the values of VIP. (FC > 1.2, *p* < 0.05, and VIP score >1); **(B)**. 20 most influential differential up-regulated and down-regulated metabolites. P values are visually represented by a color scale ranging from yellow to blue, which represents the difference from high to low. The dot sizes represent the VIP score of the metabolites. The abscissa axis is the log_2_FC, indicating the changing degree of differential metabolites.

As shown in the volcano plot, 193 differential metabolites were detected [Fold Change (FC) ≥ 1.2 or FC ≤ 0.8, *p* < 0.05, and variable importance in projection (VIP) > 1] between the control and LRM groups (Figure 5A). In the figure, each dot represents a different metabolite: green and red dots represent downregulated and upregulated metabolites respectively, and grey dots represent substandard alterations. The 20 most influential upregulated or downregulated metabolites that have been reported to possess potential biological activities were tentatively considered as biomarkers in *Caenorhabditis elegans* treated with LRM (Figure 5B; Supplementary Table S3).

#### 3.7.3 Metabolic pathway analysis

**FIGURE 6:**
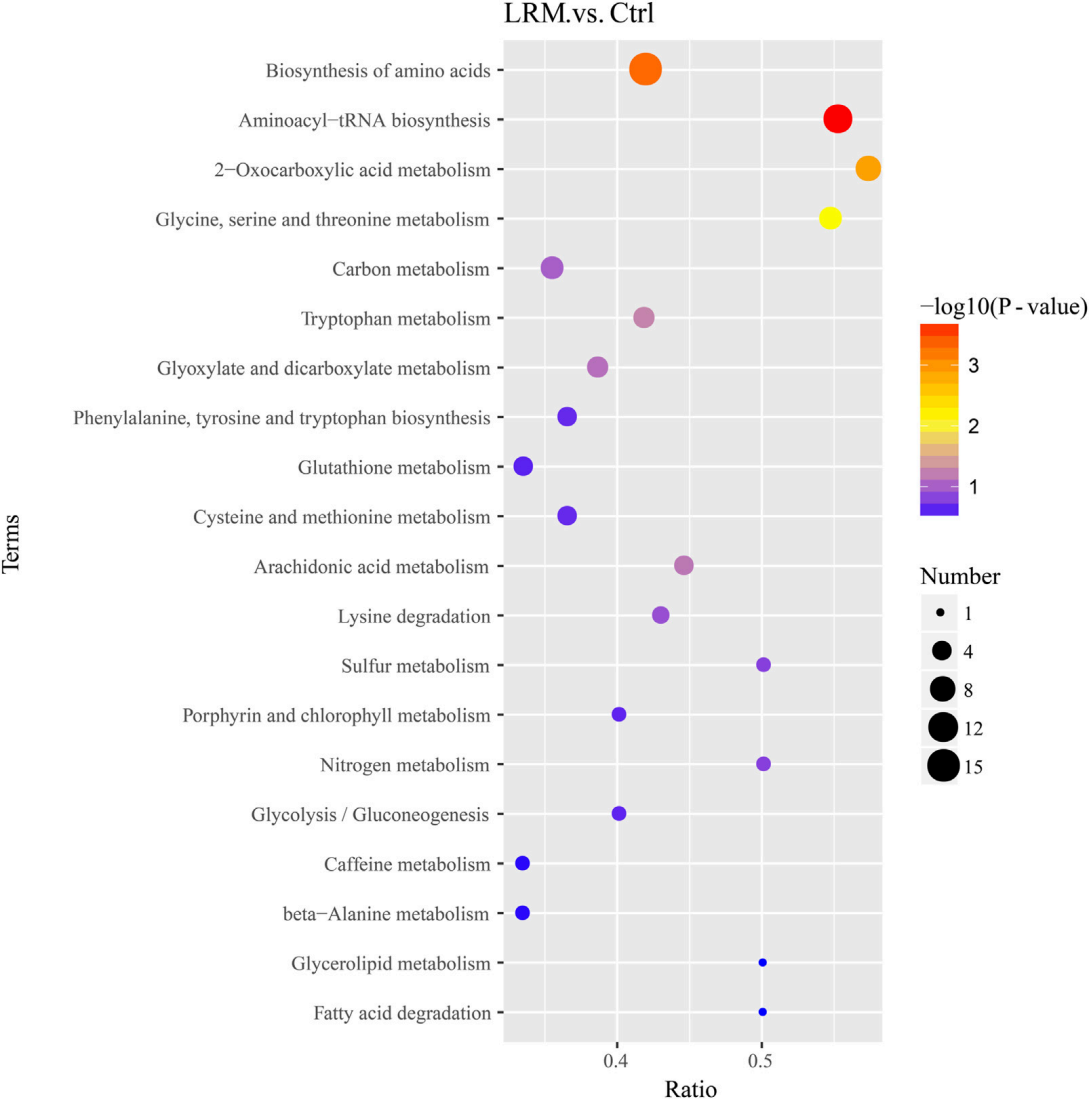
Result of top 20 metabolic pathways. P values are visually represented by a color scale ranging from red to purple, which represents the difference from high to low. The dot sizes represent the metabolite counts of the metabolic pathways. The abscissa axis is the enrichment ratio (number of differential metabolites in the corresponding metabolic pathway/total number of metabolites identified in the pathway).

Kyoto Encyclopedia of Genes and Genomes (KEGG) pathway enrichment analysis was used to analyze the differential metabolic pathways. As shown in Figure 6, the top 20 metabolic pathways in *Caenorhabditis elegans* treated with LRM were revealed in comparison with the control group. The results indicated that LRM treatment regulated 41 differential metabolic pathways, and 4 metabolic pathways were considered statistically significantly enriched, including those related to aminoacyl-tRNA biosynthesis, biosynthesis of amino acids, 2-oxocarboxylic acid metabolism, and glycine-serine-threonine (Gly-Ser-Thr) metabolism. There were 13 metabolic pathways related to lifespan in the top 20 metabolic pathways, including those associated with the biosynthesis of amino acids, 2-oxocarboxylic acid metabolism, Gly-Ser-Thr metabolism, carbon metabolism, tryptophan metabolism, glyoxylate and dicarboxylate metabolism, phenylalanine, tyrosine and tryptophan biosynthesis, glutathione metabolism, cysteine and methionine metabolism, arachidonic acid metabolism, glycolysis/gluconeogenesis, glycerolipid metabolism, and fatty acid degradation.

**FIGURE 7:**
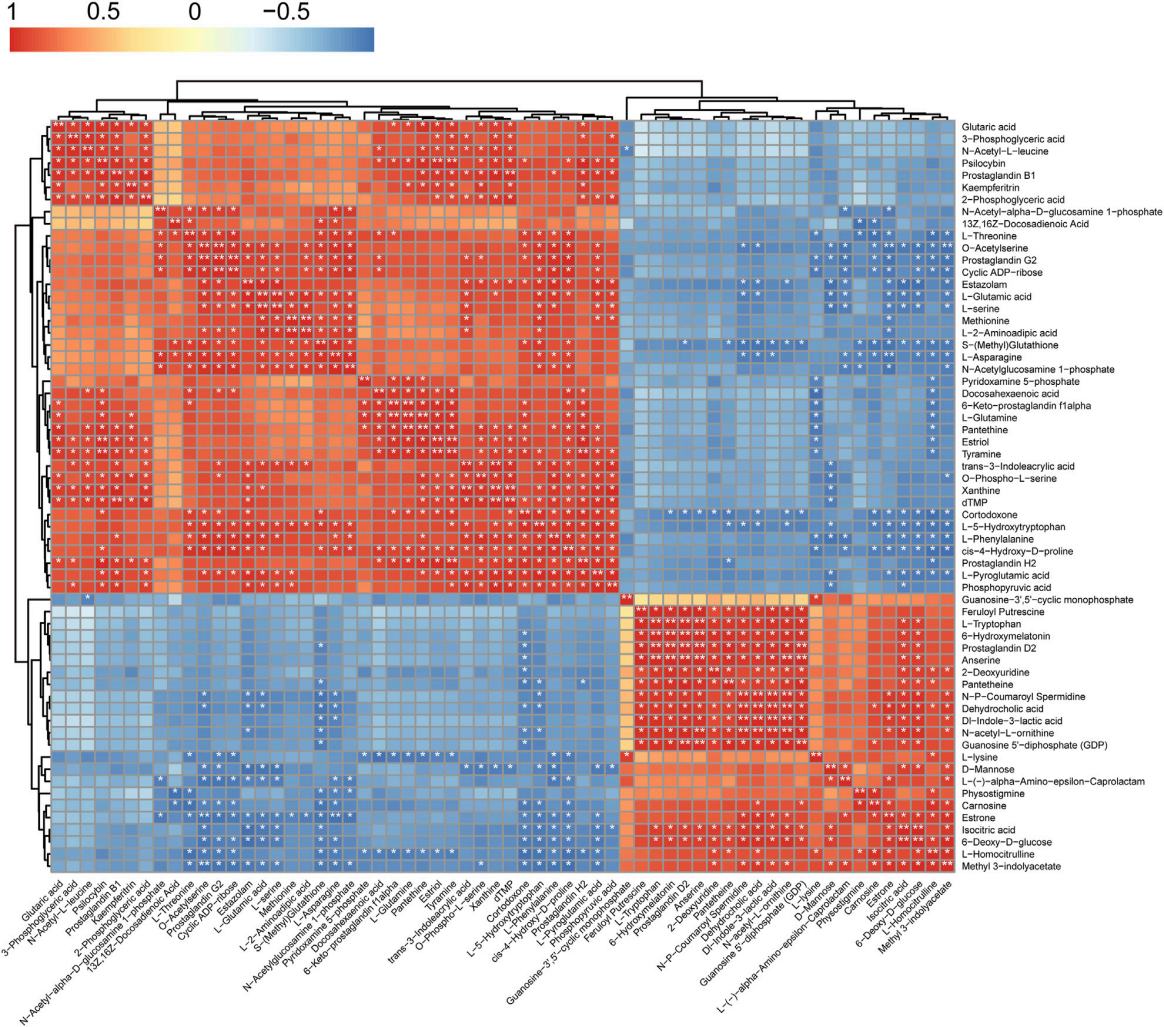
Heat map of correlations between metabolites. Each square represents the Pearson’s rank correlation coefficient between the metabolites of the row and that of the column (*, *pBH* < 0.05; **, *pBH* < 0.01).

Pearson correlation was used to analyze the relationships between differential metabolites and metabolic pathways (Pearson correlation coefficient (r) and *p*_BH_ are provided in Supplementary Tables S4, S5). Unsupervised hierarchical clustering revealed several ‘hot spots’ of highly correlated metabolites (r > 0.5; *p*_BH_ < 0.05) (Figure 7). Remarkably, *L*-serine significantly correlated with methionine, *O*-acetyl-serine, *L*-5-hydroxytryptophan, *L*-glutamic acid, *L*-asparagine, and phosphoenolpyruvate. *L*-Threonine significantly correlated with *L*-asparagine, *L*-5-hydroxytryptophan, and *O*-acetyl-serine (r > 0.9; *p*_BH_ < 0.05). These metabolites were correlated with Gly-Ser-Thr metabolism which acts as a central metabolism pathway associated with glycolysis/gluconeogenesis, glyoxylate and dicarboxylate metabolism, tryptophan metabolism, lysine degradation, sulfur metabolism, cysteine and methionine metabolism, porphyrin and chlorophyll metabolism, glutathione metabolism, and beta-alanine metabolism.

**FIGURE 8:**
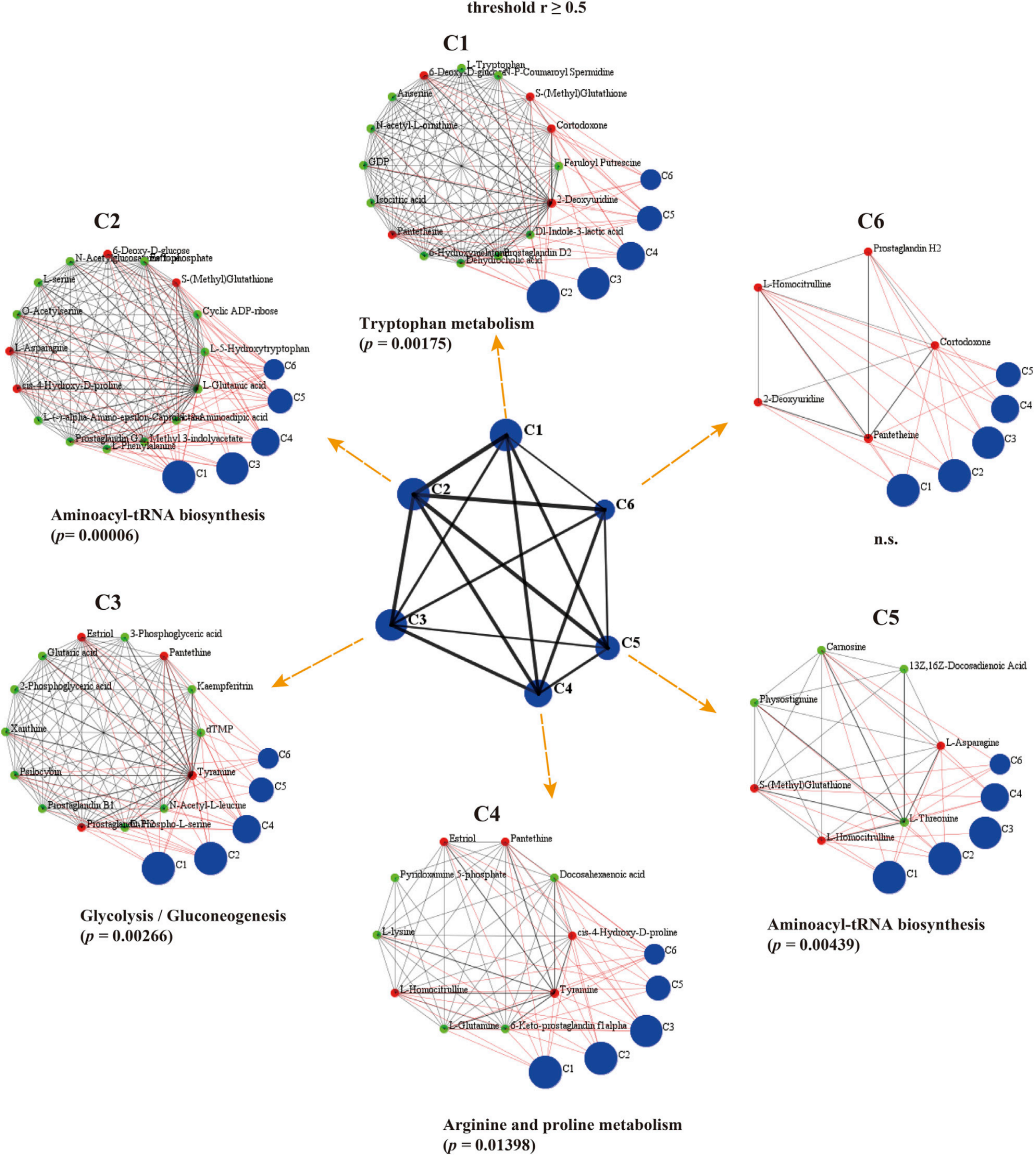
Graph clustering of metabolomic correlated modules in *Caenorhabditis elegans* with DPClusO algorithm (threshold r ≥ 0.5). The central graph displayed 6 blue clusters and 15 black edges. Each blue cluster contains closely connected metabolites labeled as C1 to 6. Small red or green nodes in the clusters represent individual metabolites. C1 to 6 show the first to sixth cluster of the hierarchical graph. The internal nodes of the clusters are connected by black edges; neighboring clusters are connected by red edges. (D = 0.9, CP = 0.5, OV Coff = 0.25).

To identify co-accumulated metabolite groups, we used simple graph clustering based on the DPClusO algorithm, an enhanced version of the DPClus algorithm that can extract densely connected nodes as a cluster (Altaf-Ul-Amin et al., 2012; Altaf-Ul-Amin et al., 2006). In previous research, the overlapping mode of the DPClus algorithm has been used to analyze the metabolic pathways (Fukushima et al., 2011). At r ≥ 0.5 *p*_BH_ < 0.05, there were 6 clusters identified by the DPClusO algorithm in the metabolomic correlation network for the *Caenorhabditis elegans* samples; they ranged in size from 5 to 16 metabolites. The KEGG enrichment analysis was used to assess the significance of the clusters (Figure 8; Supplementary Table S6). The largest cluster of C2 and C5 was “aminoacyl-tRNA biosynthesis”. These clusters may represent the extensive coordination among amino acids metabolism pathways in *Caenorhabditis elegans* treated with LRM. The largest cluster of C1 was “tryptophan metabolism,” which is connected with age-related diseases and longevity (van der Goot et al., 2012). The largest cluster of C3 was “glycolysis/gluconeogenesis”. Besides, “glycine, serine and threonine metabolism” was also significantly enriched in C3.

## 4 Discussion

Our study assessed the effect of LRM on the metabolomic of *Caenorhabditis elegans*, which revealed that even at a low concentration (5 μg/mL), LRM possesses lifespan-extending and performance-improving activities. This aligns with findings from previous research (Xiong et al., 2021). Notably, the anti-aging activity of LRM was more pronounced at lower concentrations compared to other *Lycium* spp., indicating its superior efficacy (Xiong et al., 2021). Interestingly, after treatment with LRM, pharyngeal pumping was reduced and the body length of the nematodes was shortened. Feeding-defective mutants of *Caenorhabditis elegans* such as *eat-2*, a mutant tends to have shorter body lengths and lower pharyngeal pumping rates (Avery, 1993; Morck, 2006). DR, achieved through reduced food intake or intermittent time-restricted feeding, has been proven effective in extending nematode longevity. It impacts growth signaling and resists the disruption of proteostasis (Iranon et al., 2019; Weir et al., 2017). Famous caloric-restriction mimetics, such as aspirin, resveratrol, and spermidine, may activate autophagy and prolong the lifespan without the need for calorie restrictions (Hofer et al., 2021).

Other studies have shown that natural candidates of plant origin, such as Montmorency tart cherry (*Prunus cerasus* L.) and Shatavarin IV, could have prolonged the lifespan and reduced pharyngeal pumping rates in *Caenorhabditis elegans* (van de Klashorst et al., 2020; Smita et al., 2020). Similar results were observed in LRM-treated nematodes (Xiong et al., 2021), but our study revealed an intriguing pattern: while LRM initially reduced pharyngeal pumping, it later improved pumping capacity in comparison to the control. The above results suggested that LRM induces physiological changes associated with DR and also enhances the overall health and aging process in *Caenorhabditis elegans*.

**FIGURE 9:**
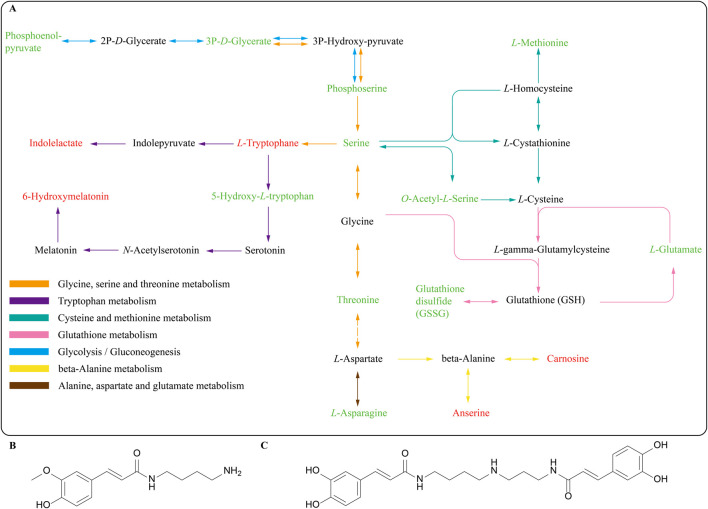
**(A)** Integration of the metabolic pathways altered by LRM treatment in *Caenorhabditis elegans*. In this path map, red represents upregulated metabolites and green represents downregulated metabolites **(B)** Feruloyl Putrescine; **(C)** N1, N10-dicaffeoyl-spermidine.

We selected metabolites related to lifespan or stress resistance from the top 20 differential metabolites for further discussion. Regulation of *L*-tryptophan, 6-hydroxy melatonin, kinetin 9-riboside, and tyramine were associated with prolonging lifespan. *L*-tryptophan, 6-hydroxy melatonin, and kinetin 9-riboside were up-regulated compared with the control group, while tyramine was down-regulated. Independent reports have indicated that the lifespans of *Caenorhabditis elegans* and *Drosophila melanogaster* extend as the ratios of Tryptophan/kynurenine increase (van der Goot et al., 2012). 6-Hydroxymelatonin has been shown to possess enhancing antioxidant activity (Perez-Gonzalez et al., 2019). Kinetin 9-riboside has been reported to possess a powerful inhibitory effect on adipogenesis (Zhang et al., 2022). A Tdc-1 mutant lacking tyramine has shown increased resistance to external stressors and prolonged lifespan in comparison to *C. elegans* (Jose De Rosa et al., 2019). Besides, the potential metabolites of LRM in *C. elegans* were found in this study. Feruloyl putrescine, a kind of polyamine, may be the metabolite of *N*1, *N*10-dicaffeoyl-spermidine in LRM (Figures 5B, 9B). Spermidine alkaloids, identified in *L. ruthenicum* and *Lycium barbarum* L. (*Solanaceae*) fruits, often contain caffeoyl or dihydrocaffeoyl moieties, while some have glycosylation (Ahad et al., 2020). Caffeoyl-spermidine derivatives from *L. barbarum* L. are considered beneficial metabolites responsible for anti-aging activities (Zhou et al., 2016). Caffeoyl-spermidine has been shown to possess inhibitory activity against SIRT1, a NAD-dependent deacetylase related to aging (Qi et al., 2018). This discovery suggests that *L. ruthenicum* spermidine may possess potential activities in the anti-aging process induced by LRM in *Caenorhabditis elegans*.

The metabolomic study revealed that LRM could regulate amino acid metabolism. Amino acids and NAD metabolism have been considered to possess a relationship with lifespan in worms, *drosophila*, and rats (Parkhitko et al., 2020). *L*-glutamate, *L*-glutamine, *L*-asparagine, *L*-threonine, *L*-serine, *O*-phospho-*L*-serine, *L*-methionine, and *L*-phenylalanine were down-regulated, while *N*-acetyl-ornithine, *L*-lysine, and *L*-tryptophan were up-regulated. We found that LRM influenced amino acid metabolism through glycine, serine, and threonine metabolism which acts as a central metabolism pathway, through KEGG analysis (Figure 9A). Gly-Ser-Thr metabolism is topologically and biochemically interconnected with numerous pathways and has been reported in mice and nematodes, as being connected with antioxidant effects and lifespan (Aon et al., 2020; Wu et al., 2021; Liu et al., 2019). A common longevity-associated pathway has been reported, which centered on Gly-Ser-Thr metabolism in mice following a caloric restriction regimen (Aon et al., 2020). These results further prove that Gly-Ser-Thr metabolism plays an important role in LRM treatment.

Tryptophan metabolism can function as a regulatory role in age-related diseases and affect longevity, which has been demonstrated in yeast, nematodes, flies, and mice (van der Goot et al., 2012). Inhibition of tryptophan degradation delayed aging in *Caenorhabditis elegans* (Oxenkrug and Navrotska, 2023). Tryptophan and indole-lactate (a kind of tryptophan metabolite) have been demonstrated to alleviate oxidative stress, inflammation, and neurodegeneration in HT-22 by H_2_O_2_ (Yin et al., 2023). Cysteine and methionine metabolism is highly correlated with oxidative stress (Bin et al., 2017). Researchers have found that methionine restriction (MetR) could stimulate the production of glutathione and reduce oxidative stress (Liu G. et al., 2017). MetR has been considered as a potential DR regimen to alleviate the unfavorable influences of DR while maintaining its positive effects (Lee et al., 2024). In our study, we also observed the downregulation of methionine and an increase in the ratio of glutathione/glutathione disulfide (GSH/GSSG) in glutathione metabolism. GSH/GSSG is a biomarker for describing cellular redox homeostasis (Enns and Cowan, 2017). Moreover, glutamate, which was annotated in glutathione metabolism, was downregulated after treatment with LRM. High concentrations of glutamate-induced oxidative damage and neurodegeneration result in a depletion of intracellular GSH (Madji et al., 2018). In beta-alanine metabolism, carnosine and anserine were upregulated. Carnosine, a natural endogenous molecule, is extensively distributed in different organisms (Caruso et al., 2021). Carnosine has an antioxidant effect, can prevent the formulation of advanced glycation end-products (AGEs), and an anti-aging activity (Caruso et al., 2021). Anserine has been shown to possess similar physiological functions to carnosine, which is a methylated form of carnosine (Boldyrev et al., 2013). Moreover, most of the amino acid changes were in good agreement with previous independent reports in the eat-2 mutant (DR model) (Gao et al., 2018).

Anthocyanins and spermidine derivatives are the major phytochemicals in LRM. A new study revealed that *L. ruthenicum* anthocyanins could prolong the lifespan via JNK-1 and DAF-16/FOXO pathways, rather than the caloric restriction pathway in *Caenorhabditis elegans* (Xu et al., 2024). However, spermidine is considered a caloric restriction mimetic. It mimics the beneficial effects of DR to prolong lifespan while limiting its negative effects (Madeo et al., 2018). The present exploration suggested that LRM could induce the DR pathway to prolong lifespan. Spermidine derivatives from *L. ruthenicum* have potential anti-aging activities via the DR pathway in *Caenorhabditis elegans*. In the future, additional research is necessary to gain a more refined understanding of the anti-aging effects of *L. ruthenicum* spermidine.

## 5 Conclusion

In this study, the anti-aging activities of LRM were investigated in *Caenorhabditis elegans*. LRM treatment not only significantly prolonged the lifespan and increased mobility but also enhanced oxidative and heat stress capacities in *Caenorhabditis elegans*. Additionally, the lipofuscin level was observed to decrease when compared with the control group. The reduction in pharyngeal pumping and shortened body length of the nematodes further supported the anti-aging effects of LRM. Moreover, the results of the metabolomic analysis suggested that LRM possessed anti-aging properties, which were mediated by regulating a series of pathways centered on glycine, serine, and threonine metabolism. The observed reduction in pharyngeal pumping and body length, the downregulation of methionine, and the regulation of Gly-Ser-Thr relevant metabolism in *Caenorhabditis elegans* provided new evidence supporting LRM’s simulation of the DR pathway to prolong lifespan. Furthermore, the significant upregulation of feruloyl putrescine might imply that caffeoyl-spermidine from *L. ruthenicum* has potential activities in the process of LRM-induced pro-longevity in *Caenorhabditis elegans*.

## Data Availability

The original contributions presented in the study are publicly available. This data can be found at MetaboLights repository, accession number MTBLS12229.
