# Supplementary material for: Anti-aging activities of an ethanolic extract of Lycium ruthenicum in Caenorhabditis elegans based on metabonomic analysis
Source: Front Pharmacol. 2025 Feb 27;16:1498280. doi: 10.3389/fphar.2025.1498280 (PMC11903438; doi:10.3389/fphar.2025.1498280)
Supplement: Supplementary file 4 [file DataSheet1.pdf]

Supplementary Material

Supplementary Figures

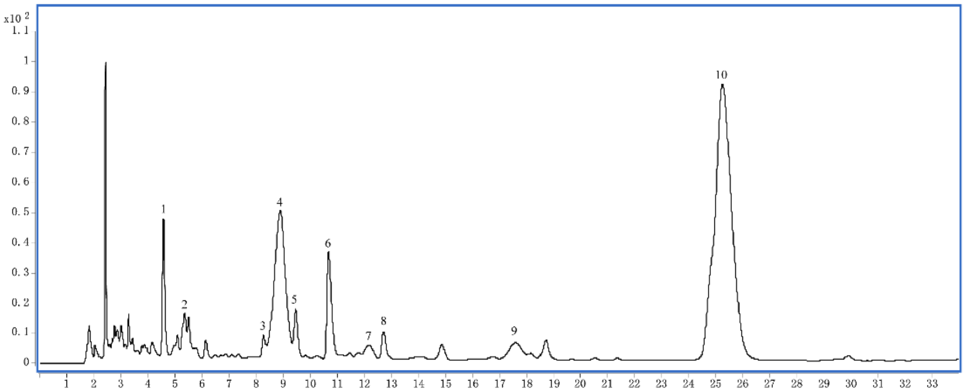

Figure S1. HPLC chromatogram of LRM at 280 nm. As shown in Figure S1 and Table S1, the main phytochemicals in LRM are anthocyanins and spermidines. Among the anthocyanins, five major compounds accounted for 99% of the total anthocyanin content. The predominant aglycone was petunidin (m/z 317), followed by delphinidin (m/z 303), which were identified by MS/MS analysis. The main glycosides were glucosides and rutinosides. Among the spermidines, lycibarbar spermidine B and N1-Dihydrocaffeoyl, N10-trans-caffeoyl-spermidine were the dominant compounds, constituting approximately 24.27% and 39.80% of the total spermidine content, respectively. These results indicate that LRM is rich in anthocyanins and spermidines, which may contribute to its biological activities.

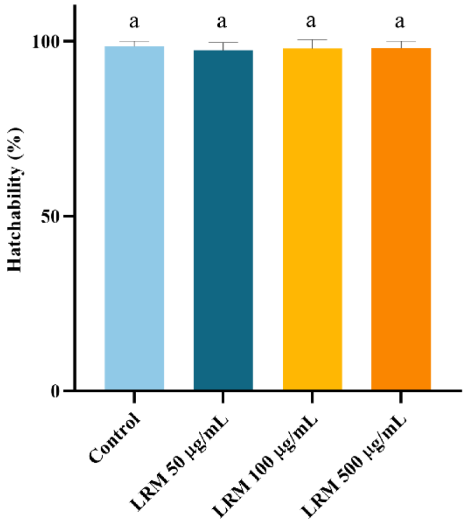

Figure S2. Effects of LRM on the incubation of C. elegans. (mean ± SEM, n = 3). Means with different superscript letters are significantly different ($p < 0.05$).

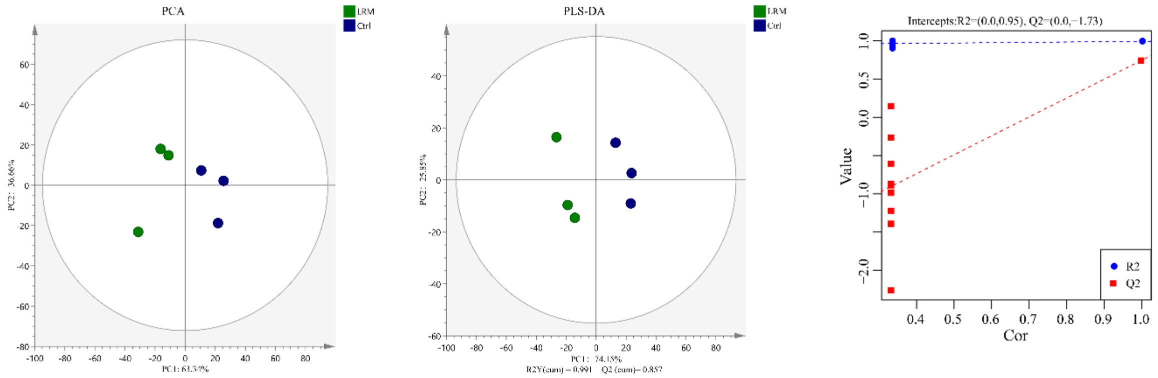

(A) **(B)** **(C)**

Figure S3. Multivariate analysis of metabolites between the control and LRM. (A) Score plot of PCA; (B) Score plot of PLS-DA; (CC) PLS-DA model validation.
